# Occurrence and Quantification of Antimicrobial Resistance Genes in the Gastrointestinal Microbiome of Two Wild Seabird Species With Contrasting Behaviors

**DOI:** 10.3389/fvets.2021.651781

**Published:** 2021-03-22

**Authors:** Ana Carolina Ewbank, Fernando Esperón, Carlos Sacristán, Irene Sacristán, Elena Neves, Samira Costa-Silva, Marzia Antonelli, Janaina Rocha Lorenço, Cristiane K. M. Kolesnikovas, José Luiz Catão-Dias

**Affiliations:** ^1^Laboratory of Wildlife Comparative Pathology, Department of Pathology, School of Veterinary Medicine and Animal Sciences, University of São Paulo, São Paulo, Brazil; ^2^Group of Epidemiology and Environmental Health, Animal Health Research Centre (INIA-CISA), Madrid, Spain; ^3^Facultad de Ciencias de la Vida, Universidad Andres Bello, Santiago, Chile; ^4^Associação R3 Animal, Florianópolis, Brazil

**Keywords:** anthropization, marine pollution, antibiotic resistance, wildlife, gull, penguin, One Health

## Abstract

Antimicrobial resistance genes (ARGs) are environmental pollutants and anthropization indicators. We evaluated human interference in the marine ecosystem through the ocurrence and quantification (real-time PCRs) of 21 plasmid-mediated ARGs in enema samples of 25 wild seabirds, upon admission into rehabilitation: kelp gull (*Larus dominicanus, n* = 14) and Magellanic penguin (*Spheniscus magellanicus, n* = 11). Overall, higher resistance values were observed in kelp gulls (non-migratory coastal synanthropic) in comparison with Magellanic penguins (migratory pelagic non-synanthropic). There were significant differences between species (respectively, kelp gull and Magellanic penguin): ARGs occurrence (*bla*_TEM_ [*p* = 0.032]; *tet*M [*p* = 0.015]; *tet*A [*p* = 0.003]; and *sul*II [*p* = 0.007]), mean number of ARGs per sample (*p* = 0.031), ARGs mean load percentage (*aad*A [*p* = 0.045], *tet*A [*p* = 0.031], *tet*M [*p* = 0.016], *bla*_TEM_ [*p* = 0.032], *sul*II [*p* = 0.008]), percentage of genes conferring resistance to an antimicrobial class (betalactams [*p* = 0.036] and sulfonamides [*p* = 0.033]), mean number of genes conferring resistance to one or more antimicrobial classes (*p* = 0.024]), percentage of multiresistant microbiomes (*p* = 0.032), and clustering (*p* = 0.006). These differences are likely due to these species' contrasting biology and ecology - key factors in the epidemiology of ARGs in seabirds. Additionally, this is the first report of *mec*A in seabirds in the Americas. Further studies are necessary to clarify the occurrence and diversity of ARGs in seabirds, and their role as potential sources of infection and dispersal within the One Health chain of ARGs.

## Introduction

Antimicrobial resistance is an issue of serious public health concern with global economic, social and political implications affecting human and animal populations, as well as the environment (1–3). This worldwide phenomenon is compromising our ability to treat infectious diseases, and undermining or preventing advances in health and medicine (4). Microbial resistance is the result of natural bacteria genetic plasticity and interactions between microbial agents, host organisms and the environment (1, 5), enhanced by the selective pressure exerted by antimicrobial usage and over-prescription in human and veterinary medicine treatments, animal and fish production (i.e., growth promoters and prophylaxis), agriculture and food technologies (1, 5, 6). The consequent remodeling of the existing microbiomes (group of all the genomic elements of a specific microbiota), associated with their dissemination capacity, confer antimicrobial resistance genes (ARGs) the role of environmental pollutants (7, 8) and indicators of environmental anthropization (2, 9, 10).

Seabirds are long-lived, wide-ranging, and upper trophic level marine predators present in all marine ecosystems and oceans of the world, from coastline to pelagic and open seas (11). By acting as predators, scavengers and cross-ecosystem nutrient ancillaries, seabirds play important roles in the processes, function and resilience of island and marine ecosystems (12). Essentially, seabirds respond rapidly to environmental changes, and due to their behavior and population dynamics, are excellent sentinels of the marine ecosystem health, reflecting natural and anthropogenic changes to the environment (13), including pollution by ARGs (14–16). In seabirds, most ARGs studies have focused on synanthropic species, due to their proximity to anthropized areas and feeding habits, and relied on classic microbiological techniques (bacterial culture and sensitivity testing) (9, 17, 18). Nevertheless, recent studies have shown that biological and ecological factors (e.g., migration and feeding niche) are also relevant to the issue of ARGs in wild birds (16, 19, 20). Additionally, only a small fraction of bacteria are cultivable (21, 22). Thus, in order to promote a more comprehensive approach, we employed highly sensitive real time polymerase chain reaction (rtPCR) methods (10, 23) to directly detect and quantify 21 selected plasmid-mediated ARGs in the gastrointestinal microbiome of two wild seabirds species (kelp gulls [*Larus dominicanus*] and Magellanic penguins [*Spheniscus magellanicus*]) upon admission to a rehabilitation center. The goals of this study were to (i) assess the presence and load of ARGs in these individuals and (ii) evaluate our findings in light of selected biological and ecological parameters (i.e., dispersal [migratory and non-migratory], feeding niche [coastal and pelagic], and interaction with human-impacted areas [synanthropic and non-synanthropic]). We hypothesized that due to their non-migratory coastal synanthropic behavior (24), kelp gull would present higher occurrence and load of ARGs than the migratory pelagic non-synanthropic Magellanic penguin (25, 26).

## Methods

### Sample Collection

Fresh fecal samples were immediately obtained by enema (16) in 25 physically restrained birds (14 kelp gulls and 11 Magellanic penguins) upon admission at the wildlife rehabilitation center (Associação R3 Animal, Florianópolis, Santa Catarina state, southern Brazil), and stored at −20°C until analyses. All birds included in the study came directly from their rescue sites (beach), and did not receive previous veterinary care prior to their arrival at the center. Total DNA extraction was carried out by a pressure filtration technique (QuickGene DNA tissue kit S, Fujifilm, Tokyo, Japan), according with the manufacturer's instructions. The 16S rRNA gene was amplified by real time PCR (rtPCR) in 10-fold dilutions of each extracted sample [(27, 28), Supplementary Materials] to verify adequate concentration of bacterial DNA. A sample was considered validated when its 10-fold dilution showed a cycle threshold (Ct) <25 (29). To normalize the study, ct was obtained based on the fluorescence variation value [(ΔF/ΔC) = 0.02] (30). Once validated, samples were analyzed by rtPCR for 21 selected ARGs encoding resistance to eight antimicrobial classes: tetracyclines (*tet*(A)*, tet*(B)*, tet*(Y)*, tet*(K)*, tet*(M)*, tet*(Q)*, tet*(S), and *tet*(W) (28), aminoglycosides [*aad*A (31) and *str* (32)], sulfonamides (*sul*I*, sul*II), chloramphenicols [*cat*I and *cat*II (28)], macrolides [*erm*(B)*, erm*(F) (33)], quinolones [*qnr*B (34) and *qnr*S (35)]; betalactams [*bla*_TEM_ (31) and *mec*A (36)], and polymyxins [*mcr-*1 (30)] (Supplementary Materials). The estimation of the percentage of bacteria harboring ARGs (mean load percentage of each ARG), was based on the formula % gene X = 10^[2+0.33(ct16S−*ctgeneX*)]^, with ct as the cycle threshold (16S rRNA regarding bacterial determination and X for each evaluated gene), and 0.33 as the mean slope for all the evaluated genes. Results were expressed in log_10_ scale of the hypothetical percentage of bacteria presenting each gene, ranging from −8 (sample considered negative) to +2 (when 100% of the bacteria in the sample presented the ARG) (30). The same thermal cycle was used for all rtPCR reactions [6′ 95°C, 40x (10″ 95°C, 30″ 60°C)], with alignment and extension in the same step, at constant 60°C. A melting curve step was performed at the end of the rtPCR reaction (30). As per (37), we applied the term “multiresistant microbiome” when a fecal sample presented at least three ARGs encoding resistance to different classes of antimicrobials (10, 29, 30). All samples used in this study were collected as part of the Santos Basin Beach Monitoring Project (Projeto de monitoramento de Praias da Bacia de Santos - PMP-BS), licensed by the Brazilian Institute of the Environment and Renewable Natural Resources (IBAMA) of the Brazilian Ministry of Environment (ABIO N° 640/2015), and in full compliance with the Biodiversity Information and Authorization System (SISBIO 59150-4). All procedures were performed according to the Ethical Committee in Animal Research of the School of Veterinary Medicine and Animal Sciences, University of São Paulo (process number 1753110716).

### Statistical Analysis

**Figure 1:**
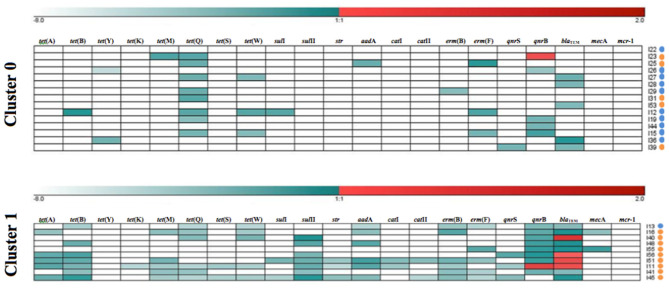
Resistance patterns of kelp gull (*Larus dominicanus*) and Magellanic penguin (*Spheniscus magellanicus*) samples obtained by *k*-means clustering of each antimicrobial resistance gene (ARG). Cluster 1 shows samples with high relative load percentage and Cluster 0 shows samples with low relative load percentage. Relative load percentage is expressed in a color scale (white for negative [−8] and dark red for the maximum value [+2]). The species are indicated on the right side (kelp gull [orange dots] and Magellanic penguin [blue dots]).

The *k*-means clustering method was used to investigate the resistance patterns (GENESIS software v. 1.7.7, Graz University of Technology, Graz, Austria), by assigning each sample to one cluster (Figure 1). Two clusters were selected, corresponding to low (value = 0) and high (value = 1) levels of ARGs. The Mann-Whitney *U* non-parametric test was used to establish the differences between species regarding: ARGs occurrence, mean number of ARGs per sample, mean load percentage of each ARG, the mean number of genes conferring resistance to one or more antimicrobial classes in each sample, percentage of multiresistant microbiomes and resistance patterns. Such statistical analyses were performed in R software (R Development Core Team 3.0.1., 2013), with a significance level of *p* < 0.05.

## Results

All the tested samples validated for the 16S rRNA gene. All animals, with the exception of one individual (96%, 24/25), were positive to at least one ARG (Table 1). ARGs results according with the species are described below.

**Table 1:** Microbiome patterns, number of detected genes per sample, and detected genes according with the animal ID and species (kelp gull *Larus dominicanus* and Magellanic penguin *Spheniscus magellanicus*).

**ID**	**Species**	**Drug class pattern**	**Number of detected ARGs**	**Detected ARGs**
I11	kelp gull	TET, SUL, AMINO, PHEN, MACR, QUINO, BLACT^†^	15	*tet*(A)*, tet*(B)*, tet*(K)*, tet*(M)*, tet*(Q)*, tet*(S), *tet*(W)*, sul*II*, str, aad*A*, cat*I*, erm*(B)*, qnr*S*, qnr*B*, bla*_TEM_
I16	kelp gull	TET, AMINO, MACR, QUINO, BLACT^†^	9	*tet*(A)*, tet*(M)*, tet*(Q)*, tet*(W)*, aad*A*, erm*(B)*, qnr*B*, bla*_TEM_, *mec*A
I23	kelp gull	TET, QUINO	3	*tet*(M)*, tet*(Q)*, qnr*B
I25	kelp gull	TET, AMINO, MACR^†^	3	*tet*(Q)*, aad*A*, erm*(F)
I56	kelp gull	TET, SUL, QUINO, BLACT^†^	6	*tet*(A)*, tet*(B)*, sul*II*, qnr*S*, qnr*B*, bla*_TEM_
I31	kelp gull	TET	1	*tet*(Q)
I39	kelp gull	QUINO, BLACT	2	*qnr*S*, bla*_TEM_
I40	kelp gull	TET, SUL, QUINO, BLACT^†^	5	*tet*(Q)*, tet*(W)*, sul*II*, qnr*B*, bla*_TEM_
I41	kelp gull	TET, SUL, MACR, QUINO, BLACT^†^	8	*tet*(B)*, tet*(M)*, tet*(Q)*, sul*II*, erm*(B)*, erm*(F)*, qnr*B*, bla*_TEM_
I45	kelp gull	TET, SUL, AMINO, PHEN, MACR, QUINO, BLACT^†^	15	*tet*(A)*, tet*(B)*, tet*(M)*, tet*(Q)*, tet*(S)*, tet*(W)*, sul*I*, sul*II*, str, aad*A*, cat*II*, erm*(B)*, erm*(F)*, qnr*S*, bla*_TEM_
I48	kelp gull	TET, SUL, AMINO, QUINO, BLACT^†^	5	*tet*(B)*, sul*II*, aad*A*, qnrB, bla*_TEM_
I51	kelp gull	TET, SUL, AMINO, PHEN, MACR, QUINO, BLACT^†^	13	*tet*(A)*, tet*(B)*, tet*(M)*, sul*I*, sul*II*, str, aad*A*, cat*I, *cat*II*,erm*(B)*, erm*(F)*, qnr*B*, bla*_TEM_
I53	kelp gull	BLACT	1	*bla*_TEM_
I55	kelp gull	MACR, QUINO, BLACT^†^	4	*erm*(F)*, qnr*B*, bla*_TEM_, *mec*A
I12	Magellanic penguin	TET, SUL, MACR^†^	5	*tet*(B)*, tet*(Q)*, tet*(W)*, sul*I*, erm*(F)
I13	Magellanic penguin	TET, AMINO, MACR, QUINO, BLACT^†^	8	*tet*(B)*, tet*(Q)*, tet*(W)*, aad*A*, erm*(B)*, erm*(F)*, qnr*B*, bla*_TEM_
I15	Magellanic penguin	TET, MACR, QUINO^†^	4	*tet*(Q)*, tet*(W)*, erm*(F)*, qnr*B
I19	Magellanic penguin	TET, QUINO	2	*tet*(Q)*, qnr*B
I22	Magellanic penguin	-	0	-
I26	Magellanic penguin	TET, QUINO	2	*tet*(Y)*, qnr*B
I27	Magellanic penguin	TET, BLACT	3	*tet*(Q)*, tet*(W)*, bla*_TEM_
I28	Magellanic penguin	BLACT	1	*bla*_TEM_
I29	Magellanic penguin	TET, MACR	2	*tet*(Q)*, erm*(B)
I36	Magellanic penguin	TET, BLACT	2	*tet*(Y)*, bla*_TEM_
I44	Magellanic penguin	QUINO	1	*qnr*B

*TET, tetracyclines; SUL, sulfonamides; AMINO, aminoglycosides; PHEN, phenicols; MACR, macrolides; QUINO, quinolone; BLACT, betalactams*.

† *Multiresistant microbiomes*.

### Kelp Gull

The *bla*_TEM_ gene presented the highest occurrence (79%, 11/14), followed by *qnr*B (64%, 9/14), *tet*(Q) (57%, 8/14), *sul*II (50%, 7/14), *tet*(B), *tet*(M), and *aad*A (43%, 6/14), *tet*(A), *erm*(B) and *erm*(F) (36%, 5/14), *tet*(W), and *qnr*S (29%, 4/29), *str* (21%, 3/21), *tet*(S)*, sul*I*, cat*I*, cat*II, and *mec*A (14%, 2/14), and *tet*(K) (7%, 1/14). The *tet*(Y) and *mcr-*1 genes were not detected in this group. The mean number of ARGs per sample was 6.4 (with min = 1 and max = 15). The *bla*_TEM_ gene presented the highest mean load percentage (−2.2) (considering ≥-3 as the median value, with −8 [min] and +2 [max]).

When clustered by antimicrobial class, kelp gulls were positive to one or more genes encoding resistance to tetracycline, quinolone and betalactams (79%, 11/14), sulfonamides and macrolides (50%, 7/14), aminoglycosides (43%, 6/14), and phenicols (21%, 3/14). No gulls presented ARGs encoding polymyxin resistance (*mcr-1*). The mean number of genes conferring resistance to one or more antimicrobial classes presented in each gull sample was four. Additionally, 71% (10/14) of the gulls presented multiresistant microbiomes (Table 1), of these, five presented two similar patterns: a tetracycline, sulfonamide, quinolone, betalactam, aminoglycoside, phenicol and macrolide combination (30%; 3/10), and a tetracycline, sulfonamide, quinolone and betalactam combination (20%; 2/10).

### Magellanic Penguin

The *tet*(Q) gene presented the highest occurrence (55%, 6/11), followed by *qnr*B (45%, 5/11), *bla*_TEM_ and *tet*(W) (36%, 4/11), *erm*(F) (27%, 3/11), *tet*(B), *tet*(Y), and *erm*(B) (18%, 2/11), *sul*I and *aad*A (9%, 1/11). Genes *tet*(A), *tet*(K)*, tet*(M), *tet*(S), *sul*II, *str, cat*I, *cat*II, *qnr*S, *mec*A, and *mcr-*1 were not detected. The mean number of ARGs per sample was 2.7 (with a maximum of eight genes per individual). Only one penguin did not present any of the tested ARGs. None of the genes presented mean load percentage ≥−3.

When clustered by antimicrobial class, individuals were positive to one or more genes encoding resistance to tetracyclines (73%, 8/11), quinolone (45%, 5/11), macrolides and betalactams (36%, 4/11), and sulfonamides and aminoglycosides (9%, 1/11). None of the individuals presented ARGs encoding chloramphenicol or polimyxin resistance. The mean number of genes conferring resistance to one or more antimicrobial classes presented in each sample was 2.1. Mutiresistant microbiomes were found in 27% (3/11) of the penguins (Table 1). Although no common patterns were observed, genes conferring resistance to tetracycline and macrolides were detected in the microbiomes of the three individuals presenting multiresistant profiles.

### Qualitative Analysis

There were significant differences between species (respectively, kelp gull and Magellanic penguin) in regards to: ARG occurrence (*bla*_TEM_ [79 and 36%. *p* = 0.032]; *tet*(M) [43 and 0%. *p* = 0.015]; *tet*(A) [36 and 0%. *p* = 0.003]; and *sul*II [50 and 0%. *p* = 0.007]), mean number of ARGs per sample (6.4 and 2.7. *p* = 0.031), ARG mean load percentage (*aad*A [−5.4 and −7.7. *p* = 0.045], *tet*(A) [−5.8 and −8. *p* = 0.031]; *tet*(M) [−5.8 and −8. *p* = 0.016]; *bla*_TEM_ [−2.2 and −5.8. *p* = 0.032]; *sul*II [−4.8 and −8. *p* = 0.008]), percentage of genes potentially conferring resistance to an antimicrobial class (betalactams [79 and 36%. *p* = 0.036] and sulfonamides [50 and 9%. *p* = 0.033]), mean number of genes conferring resistance to one or more antimicrobial classes (4 and 2.1. *p* = 0.024]), percentage of multiresistant microbiomes (71 and 27%. *p* = 0.032]), and clustering (0.6 and 0.1. *p* = 0.006]). Statistically significant differences are summarized in Table 2.

**Table 2:** Statistically significant differences between kelp gull (*Larus dominicanus*) and Magellanic penguin (*Spheniscus magellanicus*): ARG occurrence, mean number of ARGs per sample, mean load percentage of each ARG, the mean number of antimicrobial classes presented in each sample, percentage of multiresistant microbiomes, and resistance patterns.

**Parameter**	***p*-value**	**kelp gull(*n* = 14)95% CI**	**Magellanic penguin (*n* = 11) 95% CI**
Occurrence of *tet*(A)	0.03	36% (7, 64%)	0%
Occurrence of *tet*(M)	0.015	43% (13, 73%)	0%
Occurrence of *sul*II	0.007	50% (20, 80%)	0%
Occurrence of *bla*_TEM_	0.036	79% (54, 103%)	36% (2, 70%)
Mean load percentage of *tet*(A)	0.031	−5.8 (−7.6, −4.1)	−8.0
Mean load percentage of *tet*(M)	0.016	−5.8 (−7.4, −4.3)	−8.0
Mean load percentage of *sul*II	0.008	−4.8 (−6.8, −2.9)	−8.0
Mean load percentage of *aad*A	0.045	−5.4 (−7.2, −3.6)	−7.7 (−8.4, −7.0)
Mean load percentage of *bla*_TEM_	0.009	−2.2 (−4.1, −0.2)	−5.8 (−7.9, −3.7)
Percentage of resistance to sulfonamides	0.033	50% (20, 80%)	9% (−11, 29%)
Percentage of resistance to betalactams	0.036	79% (54, 103%)	36% (2, 70%)
Mean number of genes	0.031	6.4 (3.6, 9.2)	2.7 (1.2, 4.2)
Mean number of classes	0.024	4.0 (2.8, 5.2)	2.1 (1.2, 3.0)
Percentage of multiresistant microbiomes	0.032	71% (44, 98%)	27% (−4, 59%)
Clustering (0 = low; 1 = high)	0.006	0.6 (0.4, 0.9)	0.1 (−0.1, 0.3)

*Mann-Whitney U non-parametric test. Numbers in parenthesis indicate the 95% confidence interval (CI)*.

## Discussion

In accordance with our hypothesis, kelp gulls presented higher occurrence and load of ARGs than Magellanic penguins, findings that may potentially be influenced by the contrasting behaviors of these two seabird species in regard to feeding niches, interaction with human-impacted areas and dispersal. The kelp gull is the most widespread and abundant gull species in the Southern Hemisphere (38–40). Like other gull species, kelp gulls are extremely opportunistic and generalist feeders, very adapted to exploiting a wide variety of human-impacted and highly populated areas, and food subsidies (e.g., fishing discards and refuse disposals) (40–42). Such behaviors have been associated with the presence of ARGs in kelp gulls in Argentina (43), as well as in other gull species worldwide (17, 44, 45). Conversely, the Magellanic penguin is a migratory upper trophic level predator and the most abundant penguin in temperate areas, widely distributed along the southern coast of South America (24). Magellanic penguins remain in their colonies during breeding and molting periods, adopting a pelagic behavior while migrating along the continental shelf off the coast of northern Argentina, Uruguay, and southern Brazil (25, 26). Although scarce, studies on the presence of ARGs in penguins have associated ARGs occurrence with anthropization in remote locations (20, 46).

The *mec*A gene was detected in 14% (2/14) of kelp gulls, but not in penguins. This gene was reported in other wild bird groups in Brazil [passerines (47)] and Europe [corvids (48, 49), storks (50), and vultures (49)]. Nevertheless, to the best of our knowledge, this is the first report of *mec*A in seabirds in the Americas, only previously reported in European herring gulls (*Larus argentatus*) in Lithuania through metagenomics (51). The *mec*A gene is widely disseminated among *Staphylococcus aureus* and other staphylococcal species (52), encoding resistance to methicillin and cross-resistance to other β-lactam antimicrobials (52–54). Methicillin-resistant staphylococci are disseminated worldwide, frequently causing health care- and community-associated infections (52, 55), being considered one of the leading causes of nosocomial infection in Latin America (56), where it was also reported in animals, food products and the environment (57–59).

The *bla*_TEM_ gene was detected in kelp gulls and Magellanic penguins, being the most prevalent gene in the former species (79%; 11/14). *Bla*_TEM_ also presented the highest mean load percentage in this study (−2.2, in kelp gull), indicating an increased dissemination potential in comparison with the other ARGs detected here. Furthermore, the Furthermore, the *bla*_TEM_ gene presented significant differences in kelp gull in comparison with Magellanic penguin in regards to occurrence (79 and 36%. *p* = 0.032) and mean load percentage (−2.2 and −5.8. *p* = 0.032). This gene has been previously described in seabirds in Brazil (16), the United States (14, 60), and Europe Europe (51, 61–64). The TEM betalactamases confer resistance to cephalosporins and penicillins (65), one of the oldest and most widely used antimicrobial classes in humans and veterinary medicine (66, 67), partialy explaining their dissemination in the tested seabirds. Recently, a similar study in Brazil, that evaluated the microbiome of six species of wild seabirds (overall, 304 individuals), found that the *bla*_TEM_ occurrence and percentage loads ranged from 0 to 25% and −8 to −0.6, respectively, and that the *bla*_TEM_ prevalence was significantly higher in migratory in comparison with non-migratory species (16). Interestingly, despite the considerable differences regarding species and sampling size, herein we found higher *bla*_TEM_ occurrence and mean load percentages in kelp gull and Magellanic penguin, and higher *bla*_TEM_ occurrence in the non-migratory synanthropic species (kelp gull). Epidemiologically, our findings are very concerning, because while the migratory species evaluated by Ewbank et al. (16) were using a pristine habitat (Rocas Atoll), kelp gulls and Magellanic penguins are using anthropized environments. Kelp gulls especially, are using heavily anthropized areas, which likely influence not only the acquisition and potential transmission of ARGs, but also their development and maintenance, once these individuals are continuously more exposed to ARGs sources (e.g., landfills, wastewater), and consequently, to reinfection.

The genes encoding tetracycline resistance (*tet*) were the most prevalent in this study (79%; 11/14 in kelp gull and 73%; 8/11 in Magellanic penguin): *tet*(A)*, tet*(M) and *tet*(W) in kelp gull, and *tet*(Q) in Magellanic penguin. Additionally, *tet*(Q) was the most prevalent gene in the penguin group (55%, 6/11). Interestingly, Ewbank et al. (16) found that tetracycline-encoding genes were also the most prevalent antimicrobial class (ranging from 64.5 to 87.9%), significantly greater than the rest of the other ARGs classes (16). Moreover, we observed significant differences between kelp gull and Magellanic penguin in terms of *tet*(M) and *tet*(A) occurrence (43 and 0%. *p* = 0.015, and 36 and 0%. *p* = 0.003, respectively), and mean load percentage (−5.8 and −8. *p* = 0.016, and −5.8 and −8. *p* = 0.031, respectively). The high *tet* occurrence found herein was not surprising, once it had been previously detected in other seabirds in Brazil (16), and its extensive use in human and veterinary medicine, and in agriculture (68, 69). *Tet* genes have been reported in gulls in the Americas (16, 70, 71) and Europe (9, 51, 61–64, 72), and in wild penguins in Antarctica (46, 73) and Brazil (74).

Genes *sul*I and *sul*II were detected in kelp gull (*sul*II: 50% [7/14]) and in a Magellanic penguin (*sul*I: 9% [1/11]). *Sul*I and *sul*II encode resistance to sulfonamides and have been previously reported in wild seabirds in Brazil (16), with the former also reported in gulls in Europe (61–63, 72). *Sul*II presented significant differences in kelp gulls in comparison with Magellanic penguin regarding its occurrence (50% and 0%. *p* =0.007) and mean load percentage (−4.8 and −8. *p* = 0.008). Additionally, resistance to sulfonamides was significantly different in kelp gull in comparison with Magellanic penguin (50 and 9%. *p* = 0.033). Interestingly, the prevalences of sulfonamide and *sul*II gene were statistically significant higher in seabirds from an anthropized in comparison with a pristine environment (16). Sulfonamides are among the oldest synthesized antimicrobials, used in several medical therapies (75). This antimicrobial class is known to persist in the environment (76), and to resist biodegradation in wastewater-treatment processes and in media with elevated microbial activity, such as byproduct sludge (77, 78). Thus, the fact that such antimicrobial class presented more significant findings in the synanthropic coastal species (kelp gull), likely indicates higher ARGs pollution of coastal environments due to anthropogenic impact and environmental contamination (e.g., WWTP effluents and wastewater discharge) (10, 16).

Finally, we also observed significant differences in the *aad*A mean load percentage between kelp gull and Magellanic penguin (respectively, −5.4 and −7.7. *p* = 0.045). The *aad*A gene encodes resistance to two aminoglycosides: streptomycin and spectinomycin (79). Aminoglycosides are used against several aerobic Gram-negative bacilli, many staphylococci, some streptococci, and mycobacteria. Of note, streptomycin is used in multidrug treatments against multidrug-resistant *M. tuberculosis* infections (80). *Aad*A has been previously reported in gull species (61, 63, 72), and in little penguins (*Eudyptula minor*) (81).

Our findings, especially the detection of the public health relevant *mec*A and *bla*_TEM_ genes, are very concerning. The present study evaluated samples collected upon the individuals' admission into a rehabilitation center. Thus, the ARGs detected here were acquired in the wild, most likely in the environment (either in anthropized (e.g., landfills, sewage) or natural (e.g., aquatic, continental shelf) epidemiological settings), but potentially from other sources as well, such as infected food items (82) and through intra and/or interspecific interactions (e.g., kleptoparasitism). Wildlife is not naturally exposed to antimicrobial therapy in the wild, but once under treatment in rehabilitation centers, the presence of ARGs in their microbiome may interfere, and even prevent, successful therapy. Similarly to nosocomial settings, due to the intense use of antimicrobials, rehabilitation centers may be highly contaminated by these drugs and their metabolites, as well as by ARGs, and exert intense selective pressure over the local resistome (83, 84). As a consequence, rehabilitation centers may be hot spots for ARGs acquisition, interaction, and development, facilitating resistance exchanges among wildlife, humans (e.g., staff) and the environment, both while in-care and upon release (84). Thus, rehabilitation centers are very important and informative settings for the study of ARGs within the One Health interface.

Magellanic penguins are a migratory species. Bird migrations may cover great distances, through natural bio-barriers such as oceans, thus considered as holders of a potential central epidemiological role in the dissemination of ARGs, even to remote locations (3, 16, 44). Because migratory birds are capable of acquiring ARGs from humans, domestic animals and the environment (15, 17, 20, 44, 45, 85–89), this group has been largely suggested as reservoirs and dispersers of antimicrobial resistance (45, 88, 90). Despite a recent experimental study in captive ring-billed gulls (*Larus delawarensis*) in which the individuals were able to shed and contaminate the artificial environment and infect cospecifics in a controlled setting (91), further studies under natural conditions are necessary to confirm such hypothesis. Herein, migration may have not been a key factor from an epidemiological perspective of ARGs dispersal affecting humans, because despite our significant findings in Magellanic penguin [e.g., detection of ARGs in 10 out of the 11 individuals and of a gene of great public health importance (*bla*_TEM_)], this is a highly pelagic species that spends a great part of its life cycle in the oceans (26), sustaining limited direct contact with humans. By contrast, kelp gulls are not migratory, only capable of small geographical dislocations (24). Such species presents synanthropic behavior and adaptability to highly anthropized areas, in closer contact with humans and food-producing animals, consequently playing a more relevant role than Magellanic penguins in the epidemiological chain of ARGs within the human-animal-environmental interface. These findings show that all geographical dislocations – from great migrations to small geographical movements, must be considered in the study of ARGs dispersal and epidemiology.

Herein, we showed that the biological and ecological parameters evaluated in this study (i.e., dispersal [migratory and non-migratory], feeding niche [coastal and pelagic], and interaction with human-impacted areas [synanthropic and non-synanthropic]) are key factors in the complex epidemiology of ARGs in wild seabirds. Additionally, we reported the first detection of the *mec*A gene in seabirds in the Americas. Our findings greatly contribute to the current knowledge on ARGs in wild birds both nationally and worldwide, emphasize the importance of ARGs studies in wildlife rehabilitation settings, and reinforce the utility of culture-free highly sensitive molecular diagnostics to assess ARGs in the microbiome of wild birds. Nevertheless, it is important to consider the limitations of our study: (1) our techniques characterize the resistance genotype, not the phenotype, (2) microbiomes were evaluated at the exact point in time of each sample collection, and host-bacteria could eventually lose ARGs-containing plasmids prior to transmission and/or dispersal, and (3) our small sampling size. Admission and pre-release sampling and analysis would allow future assessment of rehabilitation centers as epidemiological settings. Further studies on ARGs in the microbiome of a greater number of seabirds, considering biological and ecological parameters, and the species' natural history (e.g., feeding strategy, habitat, territory), are necessary to broaden our understanding regarding the occurrence and diversity of ARGs in seabirds, and their role as potential sources of infection and dispersal within the One Health chain of ARGs acquisition, interaction, and dissemination.

## Data Availability Statement

The raw data supporting the conclusions of this article will be made available by the authors, without undue reservation.

## Ethics Statement

The animal study was reviewed and approved by the Ethical Committee in Animal Research of the School of Veterinary Medicine and Animal Sciences, University of São Paulo (process number 1753110716).

## Author Contributions

AE, FE, CS, and JC-D: conceptualization. AE, CS, IS, and EN: formal analysis. AE, CS, IS, SC-S, MA, JR, and CK: original draft preparation. All authors: review and editing.

## Conflict of Interest

The authors declare that the research was conducted in the absence of any commercial or financial relationships that could be construed as a potential conflict of interest.
