# Supplementary material for: Occurrence and Quantification of Antimicrobial Resistance Genes in the Gastrointestinal Microbiome of Two Wild Seabird Species With Contrasting Behaviors
Source: Front Vet Sci. 2021 Mar 22;8:651781. doi: 10.3389/fvets.2021.651781 (PMC8019699; doi:10.3389/fvets.2021.651781)
Supplement: Supplementary file 1 [file Table_1.DOCX]

**Supplementary table.** Primers used for real time PCR detection of selected antimicrobial resistance genes in the microbiome of kelp gull (*Larus dominicanus*) and Magellanic penguin (*Spheniscus magellanicus*).

| **Antimicrobial class** | **Gene** | **Sequence (5’-3’)** | **Sense** | **Amplicon size**  **(bp)** | **Reference** |
| --- | --- | --- | --- | --- | --- |
|  | 16S rRNA | ATGGCTGTCGTCAGCT | + | 352 | Jiang et al. 2013 |
|  |  | ACGGGCGGTGTGTAC | - |  |  |
| Aminoglycosides | *str* | AATGAGTTTTGGAGTGTCTCAACGTA | + | 147 | Wang et al. 2014 |
|  |  | AATCAAAACCCCTATTAAAGCCAAT | - |  |  |
|  | *aad*A | GCAGCGCAATGACATTCTTG | + | 282 | Devarajan et al., 2016 |
|  |  | ATCCTTCGGCGCGATTTTG | - |  |  |
| Betalactams | *bla*_TEM_ | AAAGATGCTGAAGATCA | + | 425 | Devarajan et al., 2016 |
|  |  | TTTGGTATGGCTTCATTC | - |  |  |
|  | *mec*A | CATTGATCGCAACGTTCAATTT | + | 99 | Francois et al. 2003 |
|  |  | TGGTCTTTCTGCATTCCTGGA | - |  |  |
| Macrolides | *erm*(B) | GATACCGTTTACGAAATTGG | + | 364 | Chen et al., 2007 |
|  |  | GAATCGAGACTTGAGTGTGC | - |  |  |
|  | *erm*(F) | CGACACAGCTTTGGTTGAAC | + | 309 | Chen et al., 2007 |
|  |  | GGACCTACCTCATAGACAAG | - |  |  |
| Phenicols | *cat*I | GGTGATATGGGATAGTGTT | + | 349 | Jiang et al. 2013 |
|  |  | CCATCACATACTGCATGATG | - |  |  |
|  | *cat*II | GATTGACCTGAATACCTGGAA | + | 567 | Jiang et al. 2013 |
|  |  | CCATCACATACTGCATGATG | - |  |  |
| Polymyxins | *mcr*-1 | TGATACGACCATGCTCCAAA | + | 218 | Nieto-Claudin et al., 2019 |
|  |  | GCCACCACAGGCAGTAAAAT | - |  |  |
| Quinolones | *qnr*B | GGMATHGAAATTCGCCACTG | + | 263 | Cummings et al., 2011 |
|  |  | TTYGCBGYYCGCCAGTCGAA | - |  |  |
|  | *qnr*S | GACGTGCTAACTTGCGTGAT | + | 118 | Marti and Balcázar, 2013 |
|  |  | TGGCATTGTTGGAAACTTG | - |  |  |
| Sulfonamides | *sul*I | CGCACCGGAAACATCGCTGCAC | + | 163 | Jiang et al. 2013 |
|  |  | TGAAGTTCCGCCGCAAGGCTCG | - |  |  |
|  | *sul*II | TCCGGTGGAGGCCGGTATCTGG | + | 191 | Jiang et al. 2013 |
|  |  | CGGGAATGCCATCTGCCTTGAG | - |  |  |
| Tetracycline | *tet*(A) | GCGCTNTATGCGTTGATGCA | + | 387 | Jiang et al. 2013 |
|  |  | ACAGCCCGTCAGGAAATT | - |  |  |
|  | *tet*(B) | TACGTGAATTTATTGCTTCGG | + | 206 | Jiang et al. 2013 |
|  |  | ATACAGCATCCAAAGCGCAC | - |  |  |
|  | *tet*(Y) | ATTTGTACCGGCAGAGCAAAC | + | 181 | Jiang et al. 2013 |
|  |  | GGCGCTGCCGCCATTATGC | - |  |  |
|  | *tet*(K) | TCGATAGGAACAGCAGTA | + | 169 | Jiang et al. 2013 |
|  |  | CAGCAGATCCTACTCCTT | - |  |  |
|  | *tet*(M) | ACAGAAAGCTTATTATATAAC | + | 171 | Jiang et al. 2013 |
|  |  | TGGCGTGTCTATGATGTTCAC | - |  |  |
|  | *tet*(Q) | AGAATCTGCTGTTTGCCAGTG | + | 169 | Jiang et al. 2013 |
|  |  | CGGAGTGTCAATGATATTGCA | - |  |  |
|  | *tet*(S) | GAAAGCTTACTATACAGTAGC | + | 169 | Jiang et al. 2013 |
|  |  | AGGAGTATCTACAATATTTAC | - |  |  |
|  | *tet*(W) | GAGAGCCTGCTATATGCCAGC | + | 168 | Jiang et al. 2013 |
|  |  | GGGCGTATCCACAATGTTAAC | - |  |  |

## REFERENCES

Chen, J., Yu, Z., Michel, F. C. Jr, Wittum, T., and Morrison, M. (2007). Development and application of real-time PCR assays for quantification of *erm* genes conferring resistance to macrolides-lincosamides-streptogramin B in livestock manure and manure management systems. Appl Environ Microbiol. 73(14):4407–4416. doi: 10.1128/FAEM.02799-06.

Devarajan, N., Laffite, A., Mulaji, C. K., Otamonga, J. P., Mpiana, P. T., Mubedi, J. I., et al. (2016). Occurrence of antibiotic resistance genes and bacterial markers in a tropical river receiving hospital and urban wastewaters. PloS One. 11(2):e0149211. doi: 10.1371/journal.pone.0149211.

Francois, P.; Pittet, D.; Bento, M.; Pepey, B.; Vaudaux, P.; Lew, D. et al. (2003). Rapid detection of methicillin-resistant Staphylococcus aureus directly from sterile or nonsterile clinical samples by a new molecular assay. J Clin Microbiol. 41: 254–260. doi: 10.1128/JCM.41.1.254-260.2003

Wang, F.H.; Qiao, M.; Su, J.Q.; Chen, Z.; Zhou, X. and Zhu, Y.G. (2014). High throughput profiling of antibiotic resistance genes in urban park soils with reclaimed water irrigation. Environ SciTechnol. 48: 9079-9085. [doi: 10.1021/es502615e](https://doi.org/10.1021/es502615e).
